# Binocular Perception Instance Authentication Learning for Few-Shot Visual Recognition

**DOI:** 10.3390/biomimetics11070505

**Published:** 2026-07-18

**Authors:** Chaofei Qi, Peng Li, Weiyang Lin

**Affiliations:** 1 Faculty of Computing, Harbin Institute of Technology, Harbin 150001, China; cfqi@stu.hit.edu.cn; 2 School of Mechanical Engineering and Automation, Harbin Institute of Technology Shenzhen, Shenzhen 518055, China; pl@hit.edu.cn; 3 Research Institute of Intelligent Control and Systems, Harbin Institute of Technology, Harbin 150001, China

**Keywords:** Machine visual Meta-Learning, humanoid visual meta-learning, binocular sensing, instance authentication, few-shot learning

## Abstract

Humans possess unique advantages in dealing with few-shot visual recognition scenarios, inspiring the development of meta-learning methods aimed at emulating these abilities. Current mainstream meta-learning primarily utilizes the monocular vision or the dual asymmetric complementary architectures, collectively referred to as Machine-visual Meta-Learning (MvML). Nevertheless, research has not yet developed an architecture for simulating the human binocular visual system, named Humanoid-visual Meta-Learning (HvML). This paper innovatively proposes an excellent paradigm BPIAL belonging to the HvML: Binocular Perception Instance Authentication Learning, which can alleviate the monocular shallowness and dual-branch processing instability of MvML. Structurally, our BPIAL comprises two interconnected binocular perception and information processing modules: BSEM and IAPM. The former module can simulate binocular visual field extraction, feature extraction and compression, and channel dimension reduction, while IAPM can simulate the logic, reasoning, and judgment processes of the two human visual branches and the brain. We have demonstrated the feasibility and correctness of BPIAL on five benchmarks. Sufficient and comparative experiments with the state-of-the-art methods have proved its superiority and effectiveness.

## 1. Introduction

Traditional visual recognition [1,2,3] presents quick on the uptake through constructing deep networks. However, those few-shot visual recognition scenarios normally containing fewer labeled samples sustain the extremely challenging research fields for their tendency toward over- and under-fitting. The few-shot visual recognition could be engaged with by constructing many excellent meta-learning [4,5,6,7] algorithms. To make a clear distinction, we have categorized them into two groups based on specific imaging and recognition strategies: Machine visual Meta-Learning (MvML) and Humanoid visual Meta-Learning (HvML). Ulteriorly, the former mainly focuses on how to urge learners to tackle few-shot problems according to the learned knowledge, and overlooks the deep observation and reasoning from specfic perspectives of input images (e.g, [8,9,10]). The latter aims to simulate the meta-learning principles of the human binocular visual system and construct a specific learning process similar to human multi-angle observation and logical thinking.

To systematically assess the existing MvML approaches and their limitations—especially the lack of deep observation and reasoning—we also categorize them into four types: meta-feature reconstruction, meta-network reconstruction, meta-model optimization, and meta-task metric-based. A detailed review of each category will be given below, followed by a discussion of their common deficiencies, which directly motivate our HvML framework.

The Meta-feature reconstruction-based methods focus on the post-processing of features extracted through basic encoders, rather than on the feature encoding process. These methods aim to achieve goals through learning reconstructed features, such as DPGN [11], MuL-GRN [12], Unisiam [13], ICA+MSP [14] and MixtFSL [15]. Conversely, meta-network reconstruction-based methods mainly concentrate on building novel encoders to better extract task-relevant features, thereby enhancing meta-learning performance. This kind of MvML method emphasizes the study of encoding procedures and the development of various encoding strategies to construct networks with improved generalization capabilities, such as classic methods ProtoNet [16], MatchingNet [17], and RelationNet [18], spatial embedding method FEAT [8] and attention-driven method CAN+T [19].

The meta-model optimization-based methods aim to identify better parameter optimization strategies for encoders and classifiers, with the purpose to advance the performance of meta-learning tasks. This type of MvML places greater emphasis on optimizing model parameters and seeks learning strategies that improve generalization performance while avoiding overfitting and underfitting, such as MAML [20], LEO [21], and MetaOpt [22]. The meta-task metric-based methods focus on finding appropriate similarity measures for classifying encoded features. This area of meta-learning research emphasizes the exploration of similarity or distance metrics to establish feature clustering and classification relationships, such as TADAM [23], TEAM [24], DeepEMD [25], DeepBDC [26] and others (e.g, [27]).

The meta-learning approaches discussed above are based on machine vision and avoid replicating the more comprehensive and three-dimensional feature extraction of visual information [28] that human eyes achieve. Specifically, when observing any target image, human eyes have both distinct and overlapping fields of view. Those overlapping regions enhance attention by providing a shared context, while the distinct regions can ensure individual details are preserved. Following processing by the visual system [29], the two visual branches naturally exhibit disparity, which enhances tolerance and potentially improves generalization performance. The four types of MvML all lack this characteristic, which highlights the inherent limitations of MvML compared with our proposed HvML in structure.

**Figure 1:**
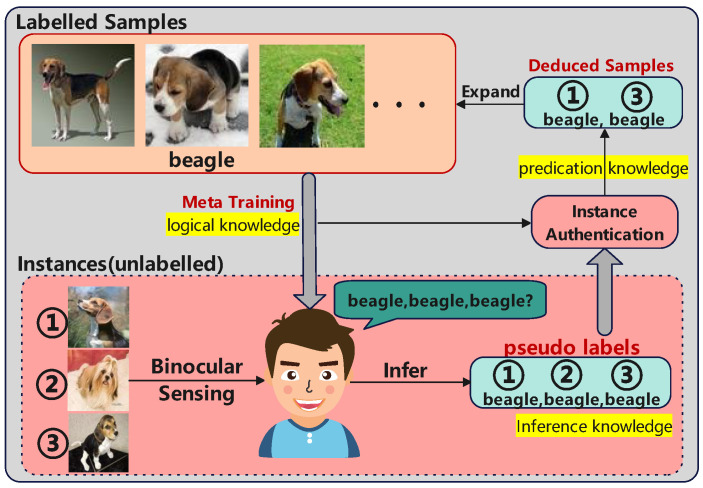
Schematic diagram of our binocular perception instance authentication learning (BPIAL). In the entire process of BPIAL, logic knowledge, inference knowledge and prediction knowledge can be learned by imitating human logic, reasoning and judgment ability, jointly promoting the final prediction results.

In this paper, we present an original paradigm of humanoid visual meta-learning: binocular perception instance authentication learning. As illustrated in Figure 1, our BPIAL can mimic humans’ eyes to capture features and infer pseudo-labels based on logical knowledge learned from meta-training. Furthermore, it deduces and selects approximately reliable pseudo-labels by binocular instance authentication, expands the labeled support set, and finally executes re-training and re-identification.

For the salient object detection scenarios [30], their original salient detection benchmarks contain not only RGB images but also an additional depth channel, which somewhat facilitates the recognition of salient regions. These RGB-D datasets provide neural networks with the inherent advantage of a human-eye perspective. However, this depth information is externally supplied by the benchmarks rather than being extracted by the networks themselves. Anyway, these networks mainly focus on highlighting salient areas, such as Refs. [31,32], and they are not meta-learning methods either. In contrast, our proposed HvML method (BPIAL) inherently simulates the binocular encoding characteristics of human vision, relying on those RGB images.

Comparatively, referring to Table 1 and taking the FEAT [8] and BML [9] (non-strict binocular networks) for example, which all belong to MvML; they both construct dual asymmetric branches (two backbones) using mutual learning strategies, respectively simulating different local and global perspectives. Distinctively, our BPIAL primitively encodes spatial and frequency domain information by constructing a symmetrical binocular structure, and simulates human binocular perception, closely mirroring the human eye recognition mechanism.

Our main contributions are summarized as follows:

- We advance an excellent natural paradigm, HvML, to engage with the few-shot learning problem by imitating human binocular observation and recognition, and propose the first meta-learning method, BPIAL, belonging to HvML.
- For binocular observation, we have proposed a complete solution realizing binocular sensing and dimension simplification: Binocular Sensing Extractor Module, BSEM.
- For logical thinking, we have proposed a complete solution simulating the human brain’s learning, reasoning, and judgment: Instance Authentication Perception Module, IAPM.
- We have verified that our BPIAL is more advantageous than MvML on five coarse- and fine-grained benchmarks.

The rest of this paper is organized as follows. In Section II, we have an in-depth discussion of the relevant literature. Then, in Section III, we introduce the principle of BPIAL and conduct modeling analysis. In Section IV, abundant comparative experiments and ablation studies are presented. Finally, we summarize the conclusion and look forward to future work.

## 2. Related Work

In order to settle few-shot image recognition problems from the perspective of instance label authentication, we introduce the representative few-shot learning methods based on MvML, embedding learning and self-taught learning for MvML.

### 2.1. Machine Visual Meta-Learning for Few-Shot Learning

Meta-learning based on meta-feature reconstruction applies a data-driven strategy, which involves data redistribution of original instances, labels, or features during the meta-training. DPGN [11] and Unisiam [13] capture intra-class commonalities and inter-class features on tasks by traversing task categories, and adopt different feature-processing strategies to boost the performance. ICA+MSP [14] and MixtFSL [15] construct the task-related metric feature subspaces based on labeled sample information, and classify the instance features extracted from backbone encoders reasonably. Meta-learning based on meta-network reconstruction employs the network-driven strategy. This approach pays more attention to the compositions of networks and constructs better meta-classifiers based on extracted features through various learning methods. For example, there exist several classical methods such as the Meta-Baseline [33], MatchingNet [17], RelationNet [18], and ProtoNet [16]. In addition, BD-CSPN [35] proposes a variety of variant networks optimized according to the prototype strategy. FEAT [8] and RENet [36] construct meta-networks by embedding extracted features into appropriate dimensional spaces. CAN+T [19] and MELR [37] introduce several attention mechanisms enhancing meta-learning ability suitable for few-shot scenarios. TDM [38] proposes a task-dependent metric module that adaptively adjusts the distance measurement based on task characteristics, achieving competitive performance on multiple benchmarks.

Meta-learning based on meta-model optimization takes the parameter optimization strategy with the aim of centering on learning to fine-tune during meta-training and meta-learns specific learnable parameters of meta classifiers, such as MAML [20]. In addition, there are some excellent implicit dual-loop optimization algorithms, such as LEO [21]. Anyway, MetaOptNet [22] is built on differentiable convex optimization and constructs explicit double-loop parameter optimization. Meta-learning founded on meta-task metrics utilizes similarity or distance measure construction strategies, aiming at learning to compare by constructing a reasonable recognition metric based on the extracted features. TADAM [23] introduces metric scaling and metric tuning into the network, and proposes a task-dependent adaptive metric. TEAM [24] constructs an adaptive metric by exploiting both the transductive learning strategy and metric learning. DeepEMD [25] utilizes the earth mover’s distance as the actual metric from the perspective of image region matching. PLCM [39] proposes pseudo-loss confidence as a metric to measure the credibility of the data through pseudo-loss distribution. DeepBDC [26] takes the Brownian distance covariance as the relative metric by combining the difference between the joint characteristic function and the edge product. Recently, several approaches such as LCCRN [40] and SRM [41] have demonstrated promising results on fine-grained few-shot benchmarks by leveraging self-attention mechanisms to capture discriminative features.

### 2.2. Embedding Learning for Machine Visual Meta-Learning

Embedding learning maps features extracted from the previous layer into a low-dimensional, dense vector space, where similar samples are closer together. This feature representation helps capture the intrinsic characteristics of few-shot samples. Generally speaking, embedding learning can significantly enhance the ability to perform effective learning and prediction in scenarios with few-shot instances through effective feature representation, similarity measurement, category generalization, and handling of data sparsity. LEO [21] aims to excavate an appropriate data-related latent feature representation of model parameters and performs gradient-based meta-learning in a relatively low-dimensional latent space to handle this problem in high-dimensional parameter spaces under extremely low data conditions. FEAT [8] employs a transformer to make the instance embedding adapt to those target few-shot recognition scenarios through a set-to-set function, and customizes a task-specific metric space through the self-attention architecture to generate a task-specific and discriminative embedding. RENet [36] applies two proposed self-correlation representation and cross-correlation attention modules to learn relationship embedding in an end-to-end manner by using relationship patterns within and between images to unravel the few-shot problem.

### 2.3. Self-Taught Learning for Machine Visual Meta-Learning

In the context of few-shot learning, self-taught learning can mitigate the challenge of limited category labels by generating relevant pseudo-labels. It extracts useful generalization information from a small number of labeled samples and leverages unlabeled data for self-optimization. This approach enhances the model’s learning effectiveness under data scarcity, thereby improving its ability to recognize unseen categories. LR+DC [42] utilizes an effective feature-distributed calibration strategy and simply constructs and trains the logistic regression with generated features, and achieving good performance without complicated generative models, training losses, and additional learning parameters. CAN [19] introduces a cross-attention module to recognize unseen classes and is repeatedly trained via self-learning. This module generates a corresponding map for each pair of class features and query instance features to highlight the target area and make the extracted features more discriminative. ICI [43] presents a linear regression hypothesis by increasing the sparsity of attached parameters to measure the credibility of all the pseudo-labels, ranks them according to their sparsity, and retrains the linear classifier with the most trustworthy pseudo-labeled instances. LR+ICI v2 [44] extends the self-taught learning paradigm by introducing an improved confidence estimation mechanism that dynamically adjusts pseudo-label selection thresholds based on task difficulty, achieving state-of-the-art performance on fine-grained few-shot benchmarks.

### 2.4. Biomimetic Vision Systems for Visual Recognition

In recent years, biomimetic vision systems have attracted increasing attention in the computer vision community. For instance, the HVNS [45] proposes a human-visual-system-inspired network that incorporates center-surround mechanisms and contrast sensitivity functions to improve feature extraction. Similarly, MUSIC [46] develops a multi-scale integration framework that mimics the hierarchical processing of the human visual cortex. These works demonstrate the potential of biologically inspired designs for visual recognition tasks. However, to the best of our knowledge, none of these approaches have been explicitly integrated with human binocular perception for few-shot scenarios, which further underscores the novelty and significance of our proposed HvML framework.

## 3. Methodology

In this section, we first analyze the few-shot visual recognition problem, elaborate on the complete operation mechanism of our BPIAL, and introduce the principle of two interconnected modules and model them: BSEM and IAPM.

### 3.1. Problem Definition

In the few-shot scenario, given a dataset $D_{\sum }$ containing $C_{\sum }$ classes to be identified, it is completely divided into three independent subsets: the base dataset $D_{base}$ with $C_{base}$ classes, the validation dataset $D_{val}$ with $C_{val}$ classes, and the novel dataset $D_{novel}$ with $C_{novel}$ classes. All of them satisfy that $D_{base}\cap D_{val}\cap D_{novel}=\;\emptyset$, and $C_{base}\cap C_{val}\cap C_{novel}=\;\emptyset$. The whole meta-learning process includes three stages: the meta-training with $D_{base}\left(C_{base}\right)$, meta-validation with $D_{val}\left(C_{val}\right)$, and meta-testing with $D_{novel}\left(C_{novel}\right)$. Each stage achieves its goal by completing the corresponding tasks of learning, which all include the support set $T_{S}={\left\{\left(X_{i},y_{i}\right)\right\}}_{i=1}^{K\times S}$ and the query set $T_{Q}={\left\{\left(X_{j},y_{j}\right)\right\}}_{j=1}^{K\times Q}$. The $T_{S}$ consists of a few labeled samples, containing *K* classes (-Way) and only S images (-Shot) per class. The $T_{Q}$ contains the same *K* classes as the support set and has Q images per class. They are both sampled from the same subset of $D_{\sum }$ and contain the same category, but the images are independent of each other and do not overlap. Typically, during meta-testing, *K* is set to 5 to facilitate the performance comparison, S to 1 or 5, and Q to 15.

### 3.2. The Mechanism of BPIAL

**Figure 2:**
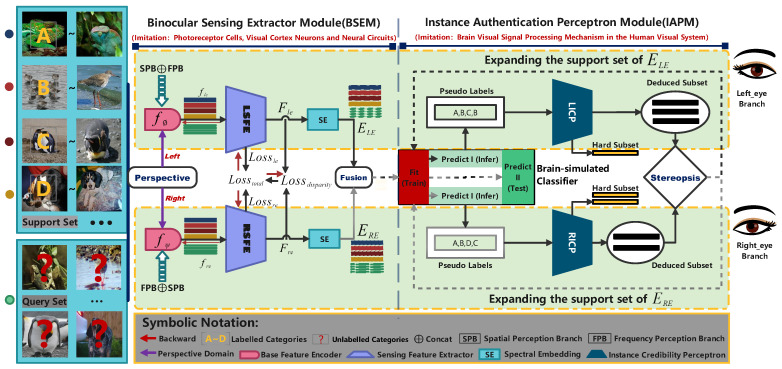
Internal structure diagram of binocular perception instance authentication learning (BPIAL). Our BPIAL consists of two relatively symmetrical branches, the Left_eye and Right_eye Branches, each of which imitates one monocular vision identification system, and they constitute the whole authentication process. LSFE and RSFE represent the left and right sensing feature extractors, and LICP and RICP represent the left and right instance credibility perceptrons, respectively. In BSEM, two encoders $f_{\phi }$ and $f_{\psi }$ process support samples to create basic features, which are then refined in LSFE and RSFE, and reduced in dimensionality via SE. In IAPM, reduced features are used to generate pseudo-labels in BsC, LICP and RICP to obtain reliable pseudo-labels, and Stereopsis is used to expand the support set. For details of the various components of the humanoid vision system, see Detail-1, Detail-2, Detail-3, Detail-4, Detail-5, Detail-6 in Section 3.

Our BPIAL framework is subdivided into two parallel visual branches according to the horizontal structure, namely the left-eye branch and the right-eye branch. They have symmetrical similar structures but are composed of components that do not share parameters, which can approximately simulate the visual field and functional similarities and differences in human eyes. From the vertical direction, the BPIAL is subdivided into two submodules: Binocular Sensing Extractor Module (BSEM) and Instance Authentication Perception Module (IAPM), which are utilized to simulate human binocular perception and instance identification. Figure 2 illustrates the specific process of BPIAL.

BSEM: Firstly, the base feature encoders $f_{\phi }$ and $f_{\psi }$ can capture and encode labeled samples from the support set and unlabeled instances from the query set, then generate two base features $f_{le}$ and $f_{re}$. The encoded base features are transmitted to the LSFE and RSFE for further analysis, and the parameters of $f_{\phi }$ and $f_{\psi }$ are continuously updated through backward propagation. We utilize spectral embedding to reduce outputs’ dimensions from SFEs and get $E_{LE}$ and $E_{RE}$, then transmit them to the Brain-simulated Classifier (BsC) after fusion.

IAPM: After fusion, input features undergo two rounds of fitting and prediction in BsC. The first fitting is mainly to fit the initial support set in two parallel branches to realize the initial shallow training of the logistic classifier. Unlabeled instances can be predicted by BsC after the first fitting; then, we obtain the pseudo-labels from both branches corresponding to them. Only reliable pseudo-labels are beneficial for BsC fitting. To further validate the generative pseudo-labels, it is essential to conduct additional analysis using LIAP and RIAP, and divide them into deduced subsets and hard subsets, respectively. By conducting stereopsis analysis on the deduced subsets $\phi_{1}$ and $\phi_{2}$, the obtained stereo set is calculated, then extended to $E_{LE}$ and $E_{RE}$. After performing quadratic fitting on the extended support, the classifier is trained in-depth twice, and then unlabeled instances are re-evaluated to produce final prediction results.

### 3.3. Binocular Sensing Extractor Module (BSEM)

Our proposed BSEM is designed to simulate the parallel process of the human eye’s double pupils and lens to capture image features and extract features through the retinas. BSEM contains the complete structure of feature encoding and extraction parts in the left and right eye branches, and its output is similar to the electrical signals after light signals are converted and processed by sensory cells in the retinas.

**Figure 3:**
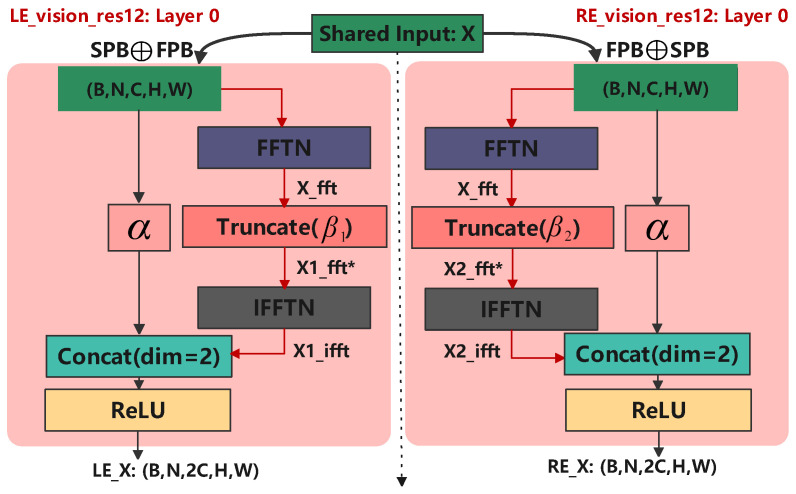
Composition diagram of the 0-th layer of Vision_Res12. The α and $\beta_{\left(.\right)}$, represent the adjustment coefficient and the frequency truncation factors, respectively. By default, α is set to constant 1, $\beta_{1}$ and $\beta_{2}$ are equal to 0.85.

Detail-1: Vision-ResNet12. To simulate the perspective of the human eyes, based on ResNet12, we supply an extra vision layer to construct Vision-ResNet12. According to the different visual fields, it can be divided into the LE_Vision_Res12 and RE_Vision_Res12, and the internal structures of their 0-th layers are illustrated in Figure 3. Given specific task *T*, we assume that the shape of the actual input stream is [B,N,C,H,W], where *B* denotes the batch size, *N* represents the total number of samples and instances in the batch, *C* indicates the image channel, and *H* and *W* denote the width and height of the sample, respectively. Given any multidimensional tensor *X* in the image stream, there are:

(1) $$\begin{array}{c} LE\text{\_}X=\mathrm{ReLU}(\alpha X⊕F^{-1}\left(T_{\beta 1}\left(F\left(X\right)\right)\right))\\ RE\text{\_}X=\mathrm{ReLU}(F^{-1}\left(T_{\beta 2}\left(F\left(X\right)\right)\right)⊕\alpha X) \end{array}$$

In the formulas, $F^{-1}$ signifies the multi-dimensional inverse fast Fourier transform, F is the multi-dimensional fast Fourier transform, $T_{\cdot }$ indicates the frequency truncation functions, ⊕ signifies the channel-wise concatenation, and the $\beta_{1}$ and $\beta_{2}$ are two independent truncation frequencies. The other neural network blocks 1-4 of LE_Vision_Res12 and RE_Vision_Res12 have the same compositions as ResNet12, and the final network output channels nFeat are identical at 512.

Here, we mark the LE_Vision_Res12 and RE_Vision_Res12 branches in our Vision-ResNet12 as $f_{\phi }$ and $f_{\psi }$, respectively. Then, we can further obtain the corresponding high-dimension and low-size feature tensors: $f_{le}=f_{\phi }^{\left(P,Q\right)}\left(X\right)$ and $f_{re}=f_{\psi }^{\left(P,Q\right)}\left(X\right)$, where $f_{le},f_{re}\in R^{B\times N\times nFeat\times P\times Q}$, and the *P* and *Q* represent corresponding height and width of final base feature maps, respectively.

Detail-2: LSFE and RSFE. To approximate the ability of the human eye to flexibly observe contours while also focusing on details, we propose two potent strategies of wide-angle attention guidance and narrow-angle attention guidance in both the LSFE and RSFE to simulate the simultaneous retraction of human eyes in sync from near to far.

Wide-angle Attention Guidance (WaAG): To have a good macroscopic view, it is indispensable to add additional layers to the original structure. Structurally, we reduce the output channel dimensions of $f_{le}$ and $f_{re}$ by adding two symmetrical but non-shared fully connected layers, and $C_{base}$ is the output channel. Let $\left(X_{i},y_{i}\right)$ be a specific image-label pair, and the WaAG output probabilities of both eyes can be expressed as:

(2) $$P_{*}^{wg}\left(y_{i}=y|X_{i}\right)=\mathrm{Softmax}\left(\sigma_{*}\left(f_{\cdot }^{\left(P,Q\right)}\left(X_{i}\right)\right)\right)$$

Here, Softmax is the activation function, symbols “·” indicate (ϕ, ψ) and “*” imply (*lr*, *re*), $\sigma_{*}$ denote two separate single-layer fully connected layers, $P^{wg}$$\in R^{B\times N\times C_{base}\times P\times Q}$, and the $P_{le}^{wg}$ and $P_{re}^{wg}$ are the actual classification prediction accuracy values from left_eye and right_eye branches, respectively.

Calculate their negative logarithm, and the losses caused by binocular wide-angle attention guidance are expressed as:

(3) $$Loss_{*}^{wg}=\underset{\left(X_{i},y_{i}\right)}{E}\frac{1}{PQ}\sum_{p=1}^{P}\sum_{q=1}^{Q}-y_{i}\log P_{*}^{wg}\left(y_{i}=y|X_{i}\right)$$

Narrow-angle Attention Guidance (NaAG). To capture suitable microscopic scenes and simulate the ability of human binoculars to directly identify details, we adopted a prototype model and proposed a new image-specific elastic constraint (IEC) based on it.

The few-shot independent samples may directly and adversely affect the prototypes while meta-training. The network’s generalization ability may be improved by appropriately scaling the distance measurement between the prototypes and the corresponding channel-level feature maps.

In detail, each monocular branch has *K* prototypes in total, which are combined as $G_{i}$, where 1≤i≤K:

(4) $$G_{i}^{*}=\frac{1}{S}\sum_{j\in T_{s}}f_{\cdot }^{\left(P,Q\right)}\left(X_{j}\right)=\frac{1}{SPQ}\sum_{j\in T_{s}}\sum_{p=1}^{P}\sum_{q=1}^{Q}f_{\cdot }^{\left(p,q\right)}\left(X_{j}\right)$$

Here, $G_{i}^{*}\in R^{B\times K\times C}$, and S denotes the total sample number of each category. We simply employ the Euclidean distance to calculate the similarity between instances and prototypes, and mark it as Euc. The *e* and $e_{\max}$ denote the current and max epochs, and γ represents a fixed adjustment factor in $d_{IEC}^{*}$ that indicates the image-specific elastic constraint terms.

(5) $$F_{\cdot }\left(X_{i}\right)=\frac{1}{PQ}\sum_{p=1}^{P}\sum_{q=1}^{Q}f_{\cdot }^{\left(p,q\right)}\left(X_{i}\right)$$

(6) $$P_{*}^{ng}\left(G_{i}^{le}=G|X_{i}\right)=\frac{\exp[-Euc\langle G_{i}^{*},F_{\cdot }\left(X_{i}\right)\rangle ]}{\sum_{j=1}^{N}\exp[-Euc\langle G_{j}^{*},F_{\cdot }\rangle ]}-\frac{e}{e_{\max}}\cdot d_{IEC}^{*}\;$$

Let $G_{P}^{*}$ represent the positive prototype of $X_{i}$, $I_{G}$ denote the G-th index in Euc〈·〉, and sort stand for positive order:

$$\varpi^{*}=Euc\langle G_{i}^{*},F_{\cdot }\rangle {|}_{I_{G}=G_{P}^{*}}-\min\left(sort\left(Euc\langle G_{i}^{*},F_{\cdot }\rangle {|}_{I_{G}\neq G_{P}^{*}}\right)\right)$$

(7) $$d_{IEC}^{*}=\frac{1}{1+\exp(-\gamma \cdot \varpi^{*})}$$

Calculate the negative logarithm, then the losses caused by the guidance of narrow-angle attention can be expressed as:

(8) $$Loss_{*}^{ng}=\underset{(X_{i},y)\in T}{E}-G_{i}^{*}\cdot \log P_{*}^{ng}\left(G_{i}^{*}=G|X_{i}\right)$$

In general, we can summarize the losses of two branches:

(9) $$Loss_{*}=Loss_{*}^{wg}+Loss_{*}^{ng}$$

Disparity. Different horizons may have different effects on final prediction results. To prevent a biggish visual deviation, the disparity between two eye branches should be considered:

Recall $f_{le}$ and $f_{re}$, and make the following representations:

(10) $$\begin{array}{c} F_{le}=f_{\phi }^{\left(P,Q\right)}\left(X_{j}\right),F_{re}=f_{\psi }^{\left(P,Q\right)}\left(X_{j}\right)\\ \Theta \langle A,B\rangle =A\cdot \log\frac{A}{B},\Omega =\frac{F_{le}+F_{re}}{2} \end{array}$$

Then, the actual disparity loss can be expressed as:

(11) $$Loss_{disparity}\left(F_{le},F_{re}\right)=\frac{1}{2}\Theta \langle F_{le},\Omega \rangle +\frac{1}{2}\Theta \langle F_{re},\Omega \rangle$$

Merging the various losses of the binocular branches, we can obtain total loss from two eyes, where ϵ is a fixed constant 1:

(12) $$Loss_{total}=Loss_{le}+Loss_{re}+\epsilon Loss_{disparity}\left(\cdot \right)$$

With backward propagation, the SGD can continuously update the model parameters to make the total loss tend to decrease.

Detail-3: Dimension Simplification. Recalling the disparity, we can obtain $\left(F_{le},F_{re}\right)$, and they are still of high dimension and small size, which need to be compressed and reduced appropriately before further logical analysis on the query instances. Here, we assume that the corresponding symbols of two-eye branch after feature compression and dimensionality reduction are $F_{\mathrm{le}}^{\prime }$ and $F_{re}^{\prime }$, $E_{LE}$ and $E_{SE}$, and the nFeat is the output dimension of the final feature maps, respectively. *B* represents the number of batches, and the total number of images contained in one complete training scenario is $I_{\sum }=B\cdot K\cdot \left(S+Q\right)$.

At this time, the compressed features can be expressed as:

(13) $$F_{*}^{\prime }=\frac{1}{P\cdot Q}\sum_{p=1}^{P}\sum_{q=1}^{Q}f_{\cdot }^{\left(p,q\right)}\left(X_{i}\right)\in R^{I_{\sum }\times nFeat}$$

Let SE[·] denote the spectral embedding method; the feature representations after dimensionality reduction are denoted as:

$$E_{LE}=SE\left[F_{le}^{\prime }\right]\in R^{I_{\Sigma }\times K_{d}},E_{RE}=SE\left[F_{re}^{\prime }\right]\in R^{I_{\Sigma }\times K_{d}},$$

$K_{d}$ represents the output dimension of spectral embedding. We then perform the feature fusion processing after embedding:

(14) $$E_{BE}=E_{LE}⊕E_{RE}\in R^{2I_{\Sigma }\times K_{d}}$$

### 3.4. Instance Authentication Perception Module (IAPM)

Detail-4: Brain-simulated Classifier (BsC). After BSEM, the results after dimensionality reduction are introduced into BsC as feature-label pairs. The multi-classification conditional probability can be written in the form of Softmax, that is

(15) $$P\left(y=j|X,\varphi \right)=\frac{e^{X\cdot \varphi_{j}}}{\sum_{i=1}^{K}e^{X\cdot \varphi_{i}}},j=1,\cdots K$$

In the above formula, $X\in E_{BE},\;\varphi_{j}\in R^{K_{d}\times K}$.

Then, the corresponding maximum likelihood function can be expressed as:

(16) $$\Gamma =\overset{2I_{\Sigma }}{\underset{i=1}{\Pi }}\overset{K}{\underset{j=1}{\Pi }}{\left(\frac{e^{X_{i}\cdot \varphi_{j}}}{\Sigma_{k=1}^{K}e^{X_{i}\cdot \varphi_{k}}}\right)}^{{\mathrm{sgn}}_{j}\left(y_{i}\right)}$$

In Equation (16), there exists an indicator function:

$${\mathrm{sgn}}_{A}\left(X\right)=\left\{\begin{array}{c} 1\;\;\;\;\;X\in A\\ 0\;\;\;\;\;X\notin A \end{array}\right.$$

Calculate the negative logarithm of Equation (16); the cost of multi-classification logarithmic probability regression is obtained:

(17) $$\mathrm{Cost}\left(\varphi \right)=-\sum_{i=1}^{2I_{\Sigma }}\sum_{j=1}^{K}{\mathrm{sgn}}_{j}\left(y_{i}\right)\ln\left(\frac{e^{X_{i}\cdot \varphi_{j}}}{\sum_{k=1}^{K}e^{X_{i}\cdot \varphi_{k}}}\right)$$

It can be made minimal by gradient descent. According to the first fitting of BsC to the support set $\left(X_{i},y\right)$, we can infer the pseudo label Y to X, and record the mean accuracy as *ℓ*.

Detail-5: LICP and RICP. With the first inference of BsC, the corresponding pseudo-labels are obtained. To further verify its effectiveness, it is necessary to perform linear analysis.

LICP and RICP have the same symmetric ICP structure. Firstly, we establish a simple linear regression model for ICP:

(18) Y=Xη+ξ

where $Y\in R^{2I_{\sum }\times K}$ and $X\in R^{2I_{\sum }\times Kd}$, η is the regression coefficient and satisfies $\eta \in R^{Kd\times K}$, ξ is the instance label intercept, and it satisfies $\xi \in R^{2I_{\sum }\times K}$.

To solve the parameter pairs (η,ξ), it is then transformed into the following optimization problem:

(19) $$\arg\min\sum_{i=1}^{2I_{\sum }}\left[\frac{1}{2}\left|\right|y_{i}-X_{i}\eta -\xi_{i}{\left|\right|}_{2}^{2}+\lambda_{1}L_{1}\left(\xi_{i}\right)+\lambda_{2}L_{2}\left(\xi_{i}\right)\right]$$

Here, both $\lambda_{1}$ and $\lambda_{2}$ are the penalty coefficients, $L_{1}\left(\xi_{i}\right)=\sum_{j=1}^{K}|\xi_{i,j}|$, and $L_{2}\left(\xi_{i}\right)=\sum_{j=1}^{K}\xi_{i,j}^{2}$.

For analysis, rewrite the above formula into matrix form:

(20) $$\left(\eta ,\xi \right)=\arg\min_{\eta ,\;\xi }\frac{1}{2}||y-X\eta -{\xi ||}_{F}^{2}+\lambda_{1}L_{1}\left(\xi \right)+\lambda_{2}L_{2}\left(\xi \right)$$

Find the partial derivative of η and let the partial derivative expansion be zero; thus, we can get:

(21) $$\eta^{*}={\left(X^{T}X\right)}^{†}X^{T}\left(Y-\xi \right)=\Lambda \left(Y-\xi \right),$$

where ${\left(\cdot \right)}^{†}$ denotes the Moore–Penrose pseudo-inverse. Bring $\eta^{*}$ into Equation (23), and we can further simplify it as:

(22) $$\arg\min_{\xi }\frac{1}{2}||Y-X\Lambda \left(Y-\xi \right)-{\xi ||}_{F}^{2}+\lambda_{1}L_{1}\left(\xi \right)+\lambda_{2}L_{2}\left(\xi \right)$$

Let $X^{*}=I-X\Lambda$ and $Y^{*}=X^{*}Y$; then, the above equation could be rewritten as:

(23) $$\xi^{*}=\arg\min_{\xi }\frac{1}{2}\left|\right|Y^{*}-X^{*}\xi {\left|\right|}_{F}^{2}+\lambda_{1}L_{1}\left(\xi \right)+\lambda_{2}L_{2}\left(\xi \right)$$

At this time, it has been transformed into the corresponding multi-response regression problem about ξ, and this optimization problem has been solved by ElasticNet. Here, assume that the input of LICP is $\left(X_{1},Y_{1}\right)$. One hundred sets of elastic regression coefficients satisfying the optimization conditions are obtained according to the regression model of LICP, and then arranged in reverse order to obtain richer features with low penalty.

With the premise of satisfying the regularization parameters minimized to the utmost, each class expands step pseudo-label samples with high confidence to form the Deduced Subset $\phi_{1}$. Suppose μ and ν represent the confidence factor and sample expansion factor of ICP, μ and ν∈[0,1], *M* = 10 is a constant:

(24) $$step=\left\{\begin{array}{c} \min(2\nu \cdot M,Q),\;\;\;if\;\ell \geq \mu \\ \max(\nu \cdot M,1),\;\;\;\;\;otherwise \end{array}\right.$$

Similarly, the Deduced Subset $\phi_{2}$ corresponding to the RICP input $\left(X_{2},Y_{2}\right)$ could also be calculated out.

Detail-6: Stereopsis Mechanism. In order to better distinguish the accuracy of the two visual branches to obtain pseudo-label samples, it is necessary to stereopsize the $\phi_{1}$ and $\phi_{2}$.

We share two simple and representative visual information processing schemes: Intersection and Union. Stereo subset ϕ of the two schemes can be calculated, and ∥ denotes *’optional’*:

(25) $$\phi =Intersection\left(\phi_{1},\phi_{2}\right)∥\;Union\left(\phi_{1},\phi_{2}\right)$$

Here, the original support set can be represented as:

$$\left(X_{S},Y_{S}\right)=\left\{\left(X_{i}{{|}_{i=1}^{KS},X_{KS+i}|}_{i=1}^{KS}\right),\left(y_{i}{{|}_{i=1}^{KS},y_{KS+i}|}_{i=1}^{KS}\right)\right\}$$

After the first prediction, the expanded support set is

(26) $$\left(X_{S}^{*},Y_{S}^{*}\right)=\left(X_{S},Y_{S}\right)+\left\{\left(X_{\phi },X_{KS+\phi }\right),\left(y_{\phi },y_{KS+\phi }\right)\right\}$$

For simplification, those feature-label pairs of the original support set can be represented as:

(27) $$\left(S_{1},S_{2}\right)={\left\{\left(X_{i},y_{i}\right),\left(X_{KS+i},y_{KS+i}\right)\right\}}_{i=1}^{K\times S}$$

In addition, the combination of query instances represents:

(28) $$X_{q}=\left(X_{q1},X_{q2}\right)={\left\{X_{j},X_{K(S+Q)+j}\right\}}_{j=KS+1}^{K\times (S+Q)}$$

Thus, relevant feature flows of left and right eye branches are:

(29) $$X_{le}=S_{1}+X_{q1},X_{re}=S_{2}+X_{q2}$$

The origin pseudo-label set of query instances is $Y_{q}=\left(Y_{q1},Y_{q2}\right)$, and the final prediction label set is $Y_{q}^{*}=\left(Y_{q1}^{*},Y_{q2}^{*}\right)$.

After stereopsis, the expanded support set is introduced into the BsC for retraining, the final prediction labels are recorded and new prediction mean accuracy $\ell^{*}$ is calculated. The complete operation scheme of our IAPM, is described in the Algorithm 1.
**Algorithm 1** Inference and Prediction Process of Our IAPM.    Input: support sample:$\left(S_{1},S_{2}\right)$, query instance:$\left(X_{q1},X_{q2}\right)$.    Initial: $\left(X_{S},Y_{S}\right)$, $X_{le},X_{re}$, $e\in \{1,...,e_{\max}\}$, *Acc* = [*ℓ*, ℓ*].    Repeat($e<=e_{\max}$):    Fit.I: Pretrain classifier BsC by using Support Set $\left(X_{S},Y_{S}\right)$.    Pred.I: Get the pseudo-label $Y_{q}$ for $X_{q}$ by BsC and cal. *ℓ*.    Rank $\left(X_{1},Y_{1}\right)=\left(X_{le},\left[Y_{S_{1}},Y_{q1}\right]\right)$ by LICP, and extract $\phi_{1}$; Rank $\left(X_{2},Y_{2}\right)=\left(X_{re},\left[Y_{S_{2}},Y_{q2}\right]\right)$ by     RICP, and extract $\phi_{2}$. Select the Stereo Subset ϕ from $\left(\phi_{1},\phi_{2}\right)$ into $\left(X_{S}^{*},Y_{S}^{*}\right)$.    Fit.II: Train classifier BsC by using Support Set $\left(X_{S}^{*},Y_{S}^{*}\right)$.    Pred.II: Get predict-label $Y_{q}^{*}$ for $X_{q}$ by BsC and cal. ℓ*.    Update e:e+1.    End Repeat

## 4. Experiments

In this part, we carry out the experiments through the following avenues: (1) compare with state-of-the-art methods on several benchmarks; (2) analyze the two branches of our BPIAL and stereopsis strategy; (3) explore the impact of the Multi-Way and Multi-Shot; (4) initialize the truncation Factors $\beta_{\left(.\right)}$ in Vision_ Res12; (5) analyze the dimension reduction and embedding method in BSEM; (6) analyze the impact of confidence ratio μ, expand ratio ν, and the number of inference iterations in IAPM on the results; (7) visualize the ablation influences of the two eye-branches and stereopsis strategies on the final predictions.

### 4.1. Datasets

To verify the efficiency of our proposed BPIAL method, we conduct extensive confirmatory experiments and ablation experiments on five publicly available few-shot recognition datasets. In the experiments, we take the average accuracy and 95% confidence interval as primary metrics, and the time difference to measure the actual speed of our algorithm’s reasoning.

CIFAR-FS [47] is short for the CIFAR100 few-shot dataset derived from CIFAR-100. It contains 100 coarse-grained categories, and each category contains almost 600 images, a total of 59,996 instances. As indicated in Table 2, CIFAR-FS is usually divided into a training subset (64 classes), a validation subset (16 classes), and a novel subset (20 classes).

The complete name of CUB-200-2011 [48] stands for the Caltech-UCSD Birds-200-2011. This is a classic bird fine-grained benchmark provided by the California Institute of Technology. This dataset contains 200 species of birds and a total of 11,788 instances. Each category includes about 60 pictures. It is usually divided into a training subset (100 classes), a validation subset (50 classes), and a novel subset (50 classes).

Aircraft-Fewshot [49] is a fine-grained classification benchmark for the few-shot scenario, which contains 100 kinds of aircraft, a total of 10,000 instances, and includes 100 images per class. It is divided into a training subset (50 classes), a validation subset (25 classes) and a novel subset (25 classes).

*Mini*ImageNet was introduced in 2016 by work [50]. The *mini*ImageNet is composed of 100 categories of images extracted from the ImageNet (ILSVRC-2012); each category contains 600 images, a total of 60,000 instances. This benchmark is usually divided into a training subset (64 classes), a validation subset (16 classes), and a novel subset (20 classes).

*Tiered*ImageNet was presented by work [51] in 2018, with 34 super-class extracted from the ImageNet ILSVRC-2012. Each super-class contains 10 to 30 sub-classes, a total of 608 sub-classes. Different from *mini*ImageNet, *Tiered*ImageNet considers the category hierarchy of ImageNet, divided into the 20 super-classes (351 sub-classes) as a training subset, 6 super-classes (97 sub-classes) as a validation subset, and 8 super-classes (160 sub-classes) as an actual novel subset.

### 4.2. Implementation Details

By and large, our experiments are divided into comparative experiments and ablation experiments. The corresponding default settings are for the comparison experiments, while the ablation experiments are adjusted accordingly on this basis. We employ the segmented SGD optimizer on the CIFAR-FS, CUB-200-2011, Aircraft-Fewshot, and *mini*ImageNet benchmarks, with the segmented learning rate set to be [(60,0.1), (80,0.006), (90,0.0012)], and the total number of epochs of meta-training is 90. For *tiered*ImageNet, we use standard SGD with the decay step-size of 30, weight decay of 5 $\times \;10^{-4}$, and set the total number of epochs to 120. During meta-training, we set 1200 batches per epoch. In the meta-validation phase or meta-testing phase, a total of 2000 iterations are performed. In Vision_Res12, we define the truncation factors $\beta_{\left(.\right)}$ to be 0.85 and the adjustment coefficient α to be constant 1. In the BSEM part, we specify the feature reduction dimension $K_{d}$ to 5, and choose spectral embedding as the embedding method. In the IAPM part, we set the confidence parameter μ to 0.5 and the expansion parameter ν to 0.5 or 0.6. During the experiments, we retain the optimal models for the 5-Way 1-Shot and 5-Shot tasks in the meta-validation phase, and finally reason them in the meta-testing phase, recording the average accuracy ±95% confidence interval as the primary evaluation metric.

### 4.3. Comparisons with the State-of-the-Arts

In Table 3, Table 4, Table 5 and Table 6, we compare the results of our BPIAL with other state-of-the-art methods on these five benchmarks. As illustrated in Table 3, our BPIAL achieves significantly more accurate results than other state-of-the-art methods of MvML on both the *mini*ImageNet and *tiered*ImageNet datasets. Compared with the transductive methods of MvML, our BPIAL can achieve excellent performance. Specifically, on *mini*ImageNet and *tiered*ImageNet, BPIAL achieves +3.18%/+2.54% higher than CAN+T, and +1.35%/+2.59% higher than MCL. Compared with current mainstream inductive state-of-the-art algorithms, our BPIAL remains able to perform superbly in terms of its superiority. In terms of mean accuracy, on *mini*ImageNet and *tiered*ImageNet, our BPIAL gets +2.02%/+2.39% higher than TDM+FRN, +3.62%/+2.83% higher than the distillation-based Rethink-Distill, +4.74%/+4.66% greater in value compared to DeepEMD, and achieves +4.50%/+2.66% superior in magnitude to the transformer algorithm – QSFormer. In Table 4, Table 5 and Table 6, our BPIAL can obtain the highest accuracy compared to SoTAs. For example, as illustrated in Table 6, our BPIAL can get +6.14%/+1.80% higher than the second-ranked TDM, and outperforms the third-ranked LCCRN by +6.60%/+2.07%, respectively. In a nutshell, our BPIAL achieves high efficiency.

### 4.4. Ablation Study

In this section, we focus on the effects of two eye branches of BPIAL and stereopsis methods on performance, the impacts of *Way* and *Shot* on results, and ablation experiments of related components in Vision-Res12, BSEM, and IAPM.

Ablation on Stereopsis, Left_eye-Branch and Right_eye-Branch. In this part, we conduct relative ablation experiments under different configurations (A), (B), (C), and (D) upon four benchmarks. Among them, the (A) and (B) represent shading the left_eye-branch and the right_eye-branch during the instance reasoning stage, respectively, and do not apply any stereopsis methods; (C) represents utilizing the Union method in the inference phase; (D) denotes our standard BPIAL using Intersection. As shown in Table 7, for 1-Shot tasks, when applying the Union as the stereopsis method, the final result is often smaller than that of a single branch; for the 5-Shot tasks, the effect of using Union is not ideal. It is apparent that the Union method may amplify uncertainties of the two eye branches, naturally increasing the difficulty to successfully identify the correct pseudo-tags in $\phi_{1}$ and $\phi_{2}$. With the Intersection, we can extract the most cross-shared trustworthy pseudo-labels from $\phi_{1}$ and $\phi_{2}$, and expand them into the support sets, which facilitates the final accuracy $\ell^{*}$ to have a stably rising trend.

Multi-Way and Multi-Shot Impact Analysis. Taking the CUB-200-2011 as an example, we analyze the influences of Multi-Ways and Multi-Shots on the accuracy and the reasoning speed of the meta-testing stage. For comparison, we replicated the LR+ICI v2 experiments on the CUB-200-2011, achieved almost identical results, and supplemented the time-consuming reasoning experiments. As shown in Table 8, for the Multi-Ways part, we set the Way value between 5 and 10, then recorded its corresponding 1-Shot and 5-Shot accuracy, and the corresponding experimental time consumption. As the class number rises, the final accuracy of inference gradually decreases. It is clear that the actual accuracy of BPIAL is about 1.5% and 0.4% higher than that of LR+ICI v2 under 1-Shot and 5-Shot, respectively, and the average time consumption is reduced by about 60%. In addition, when the Way is fixed, the 5-Shot task requires more time than the 1-Shot task. For the Multi-Shot part, we set the Shot to take six different values, then conduct all the experiments with corresponding pre-training models from 5-Way-1-Shot and 5-Way-5-Shot tasks, and meanwhile, record all the accuracy and time consumption. A similar trend also extends to Multi-Shot.

**Figure 4:**
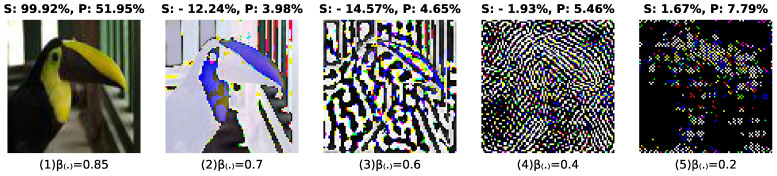
Image acquisition performance analysis with different values of truncation factors **$\beta_{\left(.\right)}$** in our Vision-ResNet12. **S** denotes SSIM, and **P** denotes PSNR.

Analysis on Truncation Factors $\beta_{\left(.\right)}$ in Vision_ResNet12. To analyze the impact of frequency domain truncation factors $\beta_{\left(.\right)}$ on images, we determine the SSIM and PSNR as essential metrics to examine the specific performance of reconstructed images obtained under different values. As illustrated in Figure 4, when $\beta_{\left(.\right)}$ are 0.85, the PSNR value on the reconstructed sample image is almost close to 50% with the premise of SSIM near 99.9%, which is in line with our expectations. When $\beta_{\left(.\right)}$ take other values, they might create a detrimental effect on SSIM, which inevitably bring more uncertainties to the downstream task. For example, when the $\beta_{\left(.\right)}$ equal 0.6, the reconstructed image has lost numerous original detail features, which would add more difficulty to the downstream image recognition tasks and increase the uncertainty of experiments. Therefore, for the sake of insurance, we define truncation factors $\beta_{\left(.\right)}$ to 0.85.

Confidence Ratio μ and Expand Ratio ν Analysis. Both μ and ν indicate the thresholds of self-learning ability. When *ℓ* is less than μ, we normally expand ν·M pseudo-labels per class into $\left(\phi_{1},\phi_{2}\right)$. However, if it is greater than μ, the number of pseudo-labels doubles, for the model has a strong recognition ability to respond to instances in the task, and increasing the number of pseudo-labels improves the model’s recognition ability for similar instances in other tasks. As illustrated in Table 9, on *tiered*ImageNet, when μ exceeds 0.7, the actual experimental accuracy is always reduced, because a larger μ means a higher requirement for *ℓ*, which is not conducive to the improvement of performance. When the value of μ is closer to or more than the algorithm’s recognition limit on the dataset, the impact will be obvious. In Table 10, we can observe that when ν is around 0.5–0.6, the best value is usually obtained, and large or too-small values have adverse effects on the BPIAL.

Reduced Dimension and Embedding Method Analysis. $K_{d}$ reflects the feature dimension of BSEM’s output embedded into BsC. The embedding method reflects the way of feature embedding. In Table 11, on *tiered*ImageNet, we conducted comparative experiments on six values of Kd and six embedding methods. Obviously, when $K_{d}$ takes 5, ℓ* reaches the maximum, and when $K_{d}$ becomes larger or smaller, it would hurt the accuracy rate. The value of $K_{d}$ has a significant impact on 1-Shot tasks and less influence on 5-Shot tasks. When the spectral embedding method is implemented, the maximum values are obtained in both 1-Shot and 5-Shot scenarios, and the second-ranked LLE also achieves relatively close accuracies.

Analysis on Inference *Iteration* of meta-testing in IAPM. The *Iteration* reflects the number of self-learning interations and the reasoning depth of the IAPM. In Table 12, on four benchmarks, we show the experimental results obtained under the four iteration values. The accuracy values (*ℓ*, ℓ*) of Predict-I and Predict-II may change slightly under different iterations. For the sake of uniformity, we define iteration = 2000 as the default setting. In addition, we can also find that the ℓ* after IAPM is several percentage points higher than *ℓ*. For example, the 1-Shot tasks of *mini*ImageNet and CUB-200-2011 improve by +6.80% and +6.88% respectively, which proves the efficiency of our IAPM.

### 4.5. Visualization

**Figure 5:**
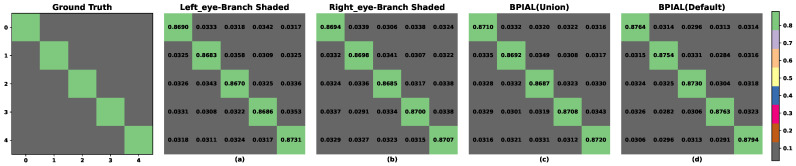
Ablation visualization of 5-Way-5-Shot classification matrices of BPIAL with different components on CIFAR-FS. (**a**) Only the Right_eye-branch is retained, (**b**) only the Left_eye-branch is retained, (**c**) BPIAL with Union, (**d**) BPIAL (default) with Intersection.

We have demonstrated the significance and indispensability of each eye branch with the ablation experiments in Table 7, and here, we will further analyze the differences in the results of these methods, where subgraphs (a–d) correspond to (A–D), respectively. In Figure 5, we visualize the detailed classification matrices obtained by different configurations of 5-Way-5-Shot tasks on CIFAR-FS. The main diagonal represents the correct recognitions of each class, and the others indicate the cases of misidentification as other classes. The accuracy results of the two monocular branches are worse than those of the binocular branches. The stereopsis method of Intersection is much more effective than the Union strategy on both correct and misclassified recognition. Doubtlessly, it also demonstrates the excellence of Intersection and the necessity of a binocular perception structure.

## 5. Conclusions and Future Work

Inspired by the principle of the human binocular vision system, we propose an original paradigm belonging to humanoid visual meta-learning (HvML), named Binocular Perception Instance Authentication Learning (BPIAL). Structurally, we propose two interconnected modules, BSEM and IAPM, which can efficiently imitate the entire procedure of human binocular logic, inference, and authentication. The primary trait of HvML includes a distinct binocular feature encoding mechanism and rich extensibility based on specific meta-learning theories. We have validated BPIAL’s superiority with numerous SoTAs on five representative benchmarks and analyzed its effectiveness through sufficient ablation experiments. In future work, we are committed to developing better mechanisms for reconstructing BSEM and IAPM, and endeavor to explore superior humanoid visual meta-learning paradigms with advanced strategies.

## Figures and Tables

**Table 1:** Comparison with several representative MvML methods on *mini*ImageNet 1-Shot Task. The light gray line represents the characteristic of our method. Obviously, our BPIAL is more effective.

Neural Network	Stage	w/Local	w/Global	MvML	HvML	Acc. (%)
ProtoNet [16]	one-stage			✔		60.37
Meta-Baseline [33]	one-stage			✔		63.17
FEAT [8]	two-stage	✔	✔	✔		66.78
BML [9]	one-stage	✔	✔	✔		67.04
MCL [10]	one-stage	✔	✔	✔		67.51
DeepEMD [34]	two-stage			✔		65.43
BPIAL (Ours)	two-stage				✔	69.91

**Table 2:** Summarization of five comparative benchmarks we used.

Benchmarks	Split-Class	Image-Size	Num./Class.
CIFAR-FS	64/16/20	84 × 84 ⇒ 84 × 84	59,996/100
CUB-200-2011	100/50/50	84 × 84 ⇒ 84 × 84	11,788/200
Aircraft-Fewshot	50/25/25	Multi-size ⇒ 84 × 84	10,000/100
*mini*ImageNet	64/16/20	Multi-size ⇒ 84 × 84	60,000/100
*tiered*ImageNet	351/97/160	224 × 224 ⇒ 84 × 84	779,165/608

**Table 3:** 5-Way result comparision of BPIAL with SoTAs on *mini*ImageNet and *tiered*ImageNet. Our results are highlighted in **bold**. The † represents deeper backbone networks than ResNet12. Our Vision-Res12 does not increase the layers with learnable parameters.

Architecture	Network	Backbone	*mini*ImageNet			*tiered*ImageNet		
			1-Shot (%)	5-Shot (%)	Mean (%)	1-Shot (%)	5-Shot (%)	Mean (%)
Machine-visual Meta-Learning (MvML)	TPN [52]	ConvNet	55.51 ± 0.86	69.86 ± 0.65	62.69 ± 0.76	59.91 ± 0.94	73.30 ± 0.75	66.61 ± 0.85
	TADAM [23]	ResNet12	58.50 ± 0.30	76.70 ± 0.30	67.60 ± 0.30	—	—	—
	DPGN [11]	ResNet12	—	—	—	72.45 ± 0.51	87.24 ± 0.39	79.85 ± 0.45
	ProtoNet [16]	ResNet12	60.37 ± 0.83	78.02 ± 0.57	69.20 ± 0.70	65.65 ± 0.92	83.40 ± 0.65	74.53 ± 0.79
	MetaOpt [22]	ResNet12	62.64 ± 0.82	78.03 ± 0.46	70.34 ± 0.64	65.99 ± 0.72	81.56 ± 0.53	73.78 ± 0.63
	LEO [21]	WRN-28 †	61.76 ± 0.08	77.59 ± 0.12	69.68 ± 0.10	66.33 ± 0.05	81.44 ± 0.09	73.89 ± 0.07
	Meta-Baseline [33]	ResNet12	63.17 ± 0.32	79.26 ± 0.17	71.22 ± 0.25	68.62 ± 0.27	83.29 ± 0.18	75.96 ± 0.23
	Unisiam [13]	ResNet18 †	64.10 ± 0.36	82.26 ± 0.25	73.18 ± 0.31	67.01 ± 0.39	84.47 ± 0.28	75.74 ± 0.34
	Rethink-Distill [53]	ResNet12	64.82 ± 0.60	82.14 ± 0.43	73.48 ± 0.52	71.52 ± 0.69	86.03 ± 0.49	78.78 ± 0.59
	ALFA+MeTAL [54]	ResNet12	66.61 ± 0.28	84.40 ± 0.44	75.51 ± 0.36	70.29 ± 0.40	86.17 ± 0.35	78.23 ± 0.38
	DeepEMD [25]	ResNet12	65.43 ± 0.28	79.28 ± 0.20	72.36 ± 0.24	69.84 ± 0.32	84.06 ± 0.23	76.95 ± 0.28
	FRN [55]	ResNet12	66.69 ± 0.19	82.89 ± 0.13	74.79 ± 0.16	71.13 ± 0.22	86.13 ± 0.15	78.63 ± 0.19
	FEAT [8]	ResNet12	66.78 ± 0.20	82.05 ± 0.14	74.42 ± 0.17	70.80 ± 0.23	84.79 ± 0.16	77.80 ± 0.20
	MCL [10]	ResNet12	67.51 ± 0.20	83.99 ± 0.20	75.75 ± 0.20	72.01 ± 0.20	86.02 ± 0.20	79.02 ± 0.20
	QSFormer [56]	ResNet12	65.24 ± 0.28	79.96 ± 0.20	72.60 ± 0.24	72.47 ± 0.31	85.43 ± 0.22	78.95 ± 0.27
	IEPT [57]	ResNet12	67.05 ± 0.44	82.90 ± 0.30	74.98 ± 0.37	72.24 ± 0.50	86.73 ± 0.34	79.49 ± 0.42
Machine-visual Meta-Learning (MvML)	CAN+T [19]	ResNet12	67.19 ± 0.55	80.64 ± 0.35	73.92 ± 0.45	73.21 ± 0.58	84.93 ± 0.38	79.07 ± 0.48
	RENet [36]	ResNet12	67.60 ± 0.44	82.58 ± 0.30	75.09 ± 0.27	71.61 ± 0.51	85.28 ± 0.35	78.45 ± 0.31
	FA-adapter [58]	ResNet12	67.79 ± 0.42	83.24 ± 0.28	75.52 ± 0.35	72.86 ± 0.50	86.60 ± 0.32	79.73 ± 0.41
	TAMF [59]	ResNet12	65.60 ± N/A	80.95 ± N/A	73.28 ± N/A	71.02 ± N/A	85.45 ± N/A	78.24 ± N/A
	FADS [60]	ResNet12	66.73 ± 0.88	83.51 ± 0.51	75.12 ± 0.70	74.12 ± 0.74	86.56 ± 0.46	80.34 ± 0.60
	HVNS [45]	ResNet12	67.30 ± 0.30	82.10 ± 0.20	74.70 ± 0.25	74.10 ± 0.40	87.40 ± 0.50	80.75 ± 0.45
	TDM+FRN [38]	ResNet12	66.96 ± 0.19	83.19 ± 0.13	75.08 ± 0.16	71.85 ± 0.22	86.55 ± 0.15	79.20 ± 0.19
HvML	BPIAL (Ours)	Vision-Res12	**69.91 ± 0.386**	**84.29 ± 0.223**	**77.10 ± 0.31**	**75.59 ± 0.421**	**87.63 ± 0.237**	**81.61 ± 0.33**

**Table 4:** 5-Way result comparision of BPIAL and SOTAs on the CUB-200-2011 benchmark. Best results are highlighted in **bold**.

Network	CUB-200-2011	
	1-Shot (%)	5-Shot (%)
IEPT [57]	69.97 ± 0.49	84.33 ± 0.33
Baseline++ [61]	67.30 ± 0.86	84.75 ± 0.60
ProtoNet [16]	66.09 ± 0.92	82.50 ± 0.58
RelationNet [18]	66.20 ± 0.99	82.30 ± 0.58
MAML [20]	67.28 ± 1.08	83.47 ± 0.59
DEML [62]	66.95 ± 1.06	77.11 ± 0.78
DeepEMD [25]	70.71 ± 0.30	86.13 ± 0.19
MELR [37]	70.26 ± 0.50	85.01 ± 0.32
QSFormer [56]	75.44 ± 0.29	86.30 ± 0.19
LCCRN [40]	82.97 ± 0.19	93.63 ± 0.10
LEADNET [63]	79.05 ± 0.20	90.85 ± 0.11
SRM [41]	84.14 ± 0.18	93.58 ± 0.09
TDM [38]	84.17 ± N/A	93.30 ± N/A
LR+ICI v2 [44]	90.38 ± 0.42	94.30 ± 0.20
BPIAL (Ours)	**92.25 ± 0.26**	**94.70 ± 0.14**

**Table 5:** 5-Way result comparision of BPIAL and SOTAs on the CIFAR-FS benchmark. Best results are highlighted in **bold**.

Network	CIFAR-FS	
	1-Shot (%)	5-Shot (%)
AFHN [64]	68.32 ± 0.93	81.45 ± 0.87
TEAM [24]	70.43 ± N/A	81.25 ± N/A
MetaOpt [22]	72.00 ± 0.70	84.20 ± 0.50
Re-Distill [53]	73.90 ± 0.80	86.90 ± 0.50
LR+ICI [43]	73.97 ± N/A	84.13 ± N/A
RENet [36]	74.51 ± 0.46	86.60 ± 0.30
TPMN [65]	75.50 ± 0.90	87.20 ± 0.60
CC+rot [66]	75.38 ± 0.31	87.25 ± 0.21
SSR [67]	76.80 ± 0.60	83.70 ± 0.40
FewTURE [68]	76.10 ± 0.88	86.14 ± 0.04
PLCM [39]	77.62 ± 1.15	86.13 ± 0.67
iLPC [69]	77.14 ± 0.95	85.23 ± 0.50
HVNS [45]	77.80 ± 0.30	87.90 ± 0.40
MUSIC [46]	77.56 ± N/A	85.49 ± N/A
BPIAL (Ours)	**79.52 ± 0.39**	**87.60 ± 0.24**

**Table 6:** 5-Way result comparision of BPIAL and SOTAs on the Aircraft benchmark. Best results are highlighted in **bold**.

Network	Aircraft-Fewshot	
	1-Shot (%)	5-Shot (%)
MixtFSL [15]	60.55 ± 0.86	77.57 ± 0.69
DeepEMD [25]	69.86 ± 0.30	85.17 ± 0.28
RelationNet [18]	74.20 ± 1.04	86.62 ± 0.55
Baseline++ [61]	74.51 ± 0.90	88.06 ± 0.44
VFD [70]	76.88 ± 0.85	88.77 ± 0.46
MatchNet [17]	82.20 ± 0.80	88.99 ± 0.50
RENet [36]	82.04 ± 0.41	90.50 ± 0.24
DeepBDC [55]	83.14 ± 0.41	93.25 ± 0.18
ProtoNet [16]	86.57 ± 0.18	93.51 ± 0.09
FRN [55]	87.53 ± 0.18	93.98 ± 0.09
MCL-Katz [10]	87.69 ± N/A	93.28 ± N/A
LCCRN [40]	88.48 ± 0.17	94.61 ± 0.08
SRM [41]	88.94 ± 0.16	94.88 ± 0.08
TDM [38]	88.35 ± 0.17	94.36 ± 0.08
BPIAL (Ours)	**95.08 ± 0.21**	**96.68 ± 0.08**

**Table 7:** Ablation study for Stereopsis methods, Left_eye-Branch and Right_eye-Branch analysis. The ■ and □ indicate whether shading the eye branch during meta-testing phase, and ■ indicates yes.

	Stereopsis	Left_Eye	Right_Eye	CIFAR-FS		*tiered*ImageNet		CUB-200-2011		*mini*ImageNet	
				1-Shot	5-Shot	1-Shot	5-Shot	1-Shot	5-Shot	1-Shot	5-Shot
(A)	*N/A*	■	□	77.77	86.96	74.69	87.15	91.78	94.47	68.60	82.73
(B)	*N/A*	□	■	77.21	86.91	75.04	87.19	91.91	94.57	69.55	83.27
(C)	*Union*	□	□	76.73	87.03	73.67	87.25	91.13	94.40	68.00	83.04
(D)	*Intersection*	□	□	**79.52**	**87.60**	**75.59**	**87.63**	**92.25**	**94.70**	**69.91**	**84.29**

**Table 8:** The ablation study of *Ways* and *Shots* on CUB-200-2011 in the meta-testing stage. The **‡** denotes the results we reproduce. The **.1** and **.5** represent the ablation results of *Ways* with 1-Shot and 5-Shot, respectively. In addition, the .♪ and .♫ represent the ablation results of *Shots* based on the 5-Way-1-Shot and 5-Way-5-Shot pre-trained models, respectively.

Network	Index	nKnovel						Index	nExemplars					
		Way = 5	Way = 6	Way = 7	Way = 8	Way = 9	Way = 10		Shot = 1	Shot = 5	Shot = 10	Shot = 15	Shot = 20	Shot = 25
ICI v2 ‡	Acc.1	90.96	89.43	86.93	84.77	83.33	81.31	Acc.♪	90.96	94.37	94.82	94.99	95.28	95.44
	Acc.5	94.39	93.44	92.33	91.41	90.38	89.55	Acc.♫	90.88	94.39	94.90	95.20	95.40	95.64
	Time.1	21 min	86 min	123 min	124 min	133 min	138 min	Time.♪	26 min	47 min	61 min	83 min	98 min	118 min
	Time.5	27 min	103 min	126 min	127 min	137 min	141 min	Time.♫	23 min	47 min	60 min	83 min	101 min	119 min
BPIAL	Acc.1	92.25	90.84	88.38	86.69	85.22	83.70	Acc.♪	92.25	95.20	95.52	95.71	95.83	96.07
	Acc.5	94.70	93.77	92.63	91.75	90.64	89.87	Acc.♫	91.09	94.70	95.10	95.36	95.46	95.70
	Time.1	15 min	36 min	45 min	48 min	53 min	58 min	Time.♪	20 min	40 min	48 min	65 min	66 min	67 min
	Time.5	18 min	46 min	50 min	66 min	76 min	78 min	Time.♫	19 min	40 min	45 min	64 min	66 min	67 min
ΔTime ↓	ΔTime.1	6 min	50 min	88 min	76 min	80 min	80 min	ΔTime.♪	6 min	7 min	13 min	18 min	32 min	51 min
	ΔTime.5	9 min	57 min	76 min	61 min	61 min	63 min	ΔTime.♫	4 min	7 min	15 min	19 min	35 min	52 min

**Table 9:** Ablation study for confidience ratio (μ) analysis on three benchmarks with ν = 0.5.

μ	*tiered*ImageNet		CUB-200-2011		Aircraft-Fewshot	
	1-Shot	5-Shot	1-Shot	5-Shot	1-Shot	5-Shot
0.8	73.39	87.34	91.78	94.59	95.02	96.61
0.7	74.41	87.59	92.16	94.61	95.08	96.61
0.6	75.23	87.62	92.25	94.61	95.08	96.61
0.5	75.57	87.63	92.24	94.61	95.08	96.61
0.4	75.59	87.63	92.24	94.61	95.08	96.61
0.3	75.57	87.63	92.24	94.61	95.08	96.61
0.2	75.58	87.63	92.24	94.61	95.08	96.61

**Table 10:** Ablation study for expand ratio (ν) analysis on three dataset benchmarks with μ = 0.5.

ν	*tiered*ImageNet		CUB-200-2011		Aircraft-Fewshot	
	1-Shot	5-Shot	1-Shot	5-Shot	1-Shot	5-Shot
0.8	73.66	87.08	90.81	94.39	94.71	96.59
0.7	74.30	87.39	91.32	94.60	94.95	96.68
0.6	75.17	87.67	91.99	94.70	95.05	96.66
0.5	75.59	87.63	92.24	94.61	95.08	96.61
0.4	75.63	87.34	92.17	94.40	95.02	96.49
0.3	74.22	86.96	91.12	94.17	94.36	96.42
0.2	70.66	86.34	87.60	93.79	92.63	96.29

**Table 11:** Ablation study for multiple reduced dimention and different embbeding methods analysis in BSEM on *tiered*ImageNet.

$K_{d}$	1-Shot Acc.	5-Shot Acc.	Emb.M.	1-Shot Acc.	5-Shot Acc.
2	74.03 ± 0.408	86.95 ± 0.238	MDS	72.37 ± 0.404	86.64 ± 0.243
5	75.59 ± 0.421	87.63 ± 0.237	PCA	75.42 ± 0.419	87.52 ± 0.236
10	75.10 ± 0.416	87.60 ± 0.238	ISOMAP	75.33 ± 0.416	87.45 ± 0.237
20	74.58 ± 0.413	87.32 ± 0.239	LTSA	74.29 ± 0.418	87.37 ± 0.236
50	72.91 ± 0.404	86.89 ± 0.241	LLE	75.58 ± 0.417	87.53 ± 0.235
64	72.23 ± 0.400	86.68 ± 0.243	SE	75.59 ± 0.421	87.63 ± 0.237

**Table 12:** Ablation study for the meta-testing *Iteration* of IAPM analysis.

*Iteration*	*mini*ImageNet 1-Shot			Aircraft 5-Shot			CUB-200-2011 1-Shot			*tiered*ImageNet 5-Shot		
	I (*ℓ*)	II (ℓ*)	Boost	I (*ℓ*)	II (ℓ*)	**Boost**	I (*ℓ*)	II (ℓ*)	Boost	I (*ℓ*)	II (ℓ*)	Boost
500	63.00	69.62	6.62 ↑	95.70	96.48	0.78 ↑	85.62	92.20	6.58 ↑	84.71	87.86	3.15↑
1000	62.92	69.68	6.76 ↑	95.77	96.59	0.82 ↑	85.69	92.42	6.73 ↑	84.39	87.46	3.07 ↑
1500	63.10	69.97	6.87 ↑	95.82	96.66	0.84 ↑	85.59	92.41	6.82 ↑	84.60	87.65	3.05 ↑
2000	63.11	69.91	6.80 ↑	95.87	96.68	0.81 ↑	85.47	92.25	6.88 ↑	84.56	87.63	3.07 ↑

## Data Availability

The data is available at https://github.com/ChaofeiQI/BPIAL (accessed on 14 July 2026).
